# Interdisciplinary approaches to understand traumatic stress as a public health problem

**DOI:** 10.1080/20008198.2018.1441582

**Published:** 2018-03-08

**Authors:** Paul Frewen, Christian Schmahl, Miranda Olff

**Affiliations:** a Department of Psychiatry, Western University, London, Canada; b Department of Psychosomatic Medicine and Psychotherapy, Central Institute of Mental Health, Mannheim, Germany; c Academic Medical Center at the University of Amsterdam, and Arq Psychotrauma Expert Group, The Netherlands

**Keywords:** posttraumatic stress disorder (PTSD), trauma, public health, innovative technologies

## Abstract

In November 2016 researchers, clinicians, and student experts gathered in Dallas, Texas, USA for the annual meeting of the International Society for Traumatic Stress Studies (ISTSS). From the description of the meeting theme forward it was acknowledged that interdisciplinary approaches will be required to fully understand traumatic stress as a public health problem, including epidemiology, biostatistics and health services research. Further, it was recognized that knowledge translation would be of critical importance if we were to increase public awareness of - and destigmatize - posttraumatic biopsychosocial problems, including posttraumatic stress disorder (PTSD). Moreover, we recognized innovative technologies as potentially usefully applied to public health strategies, including media and internet usage might aid knowledge translation and have a direct impact on help-seeking and trauma-informed care. The present special issue of the journal brings a collection of papers from some of the most impactful and innovative ideas that were presented in Dallas.

## Abordajes interdisciplinarios para entender el estrés traumático como un problema de salud pública

En noviembre de 2016, expertos investigadores, clínicos y estudiantes se reunieron en Dallas, Texas, EE. UU. para la reunión anual de la*International Society for Traumatic Stress Studies *(ISTSS; Sociedad Internacional de Estudios de Estrés Traumático). La propia descripción del tema de la reunión reconoce que son necesarios abordajes interdisciplinarios para comprender plenamente el estrés traumático como un problema de salud pública, que incluya la epidemiología, la bioestadística y la investigación de los servicios de salud. Además, se reconoció que sería de suma importancia traducir los conocimientos para concienciar al público sobre los problemas biopsicosociales postraumáticos, incluido el trastorno de estrés postraumático (TEPT), y desestigmatizarlos. Además, reconocimos que las tecnologías innovadoras se pueden aplicar de manera potencialmente útil a las estrategias de salud pública, incluidos los medios de comunicación y el uso de Internet, y que pueden ayudar a traducir los conocimientos y tener un impacto directo en la búsqueda de ayuda y la atención clínica basada en el trauma. Este número especial de la revista incorpora una colección de artículos sobre algunas de las ideas más impactantes e innovadoras que se presentaron en Dallas.

## 用跨学科方法理解作为公众健康问题的创伤应激

在2016年11月，研究者、临床医生、学生、专家在美国德克萨斯州达拉斯聚集，参加了国际创伤应激研究协会（ISTSS）的年度会议。会议的主题说明，要全面理解作为公众健康问题的创伤应激，需要使用跨学科的方法，包括流行病学、生物统计和健康服务研究。并且，如果我们要提高公众对创伤后的生理心理社会问题（包括 PTSD）的认识，并做到去污名化，应该意识到知识转化的重要性。另外，我们意识到创新的技术可能作为公众健康策略发挥用途，包括媒体和网络使用都有可能支持知识转化，并直接影响帮助寻求和创伤护理。这本特刊包含了一系列的文章，它们来自于在达拉斯呈现过的最优影响力和创新性的想法中的一些。

In November 2016, over 1100 researchers, clinicians and student experts gathered in Dallas, Texas, U.S.A. for the annual meeting of the International Society for Traumatic Stress Studies (ISTSS). ISTSS Scientific Committee Co-Chairs Dr Schmahl and Dr Frewen, with then ISTSS president Dr Greta Dyb, sought to convene a meeting with the primary goal to recognize traumatic stress as a global public health problem (Magruder, McLaughlin, & Elmore Borbon, 2017). From the description of the meeting theme forward, it was acknowledged that interdisciplinary approaches will be required to fully understand traumatic stress as a public health problem, including epidemiology, biostatistics and health services research. Further, it was recognized that knowledge translation would be of critical importance if we were to increase public awareness of – and destigmatize – post-traumatic biopsychosocial problems, including post-traumatic stress disorder (PTSD) and other trauma- and stressor-related disorders. Moreover, we recognized innovative technologies as potentially usefully applied to public health strategies, where modern technologies including media and Internet usage might aid knowledge translation and have a direct impact on help-seeking and trauma-informed care. Modern technologies were also recognized as potentially directly advancing assessment, treatment and prevention efforts. As has become a good tradition, (e.g. Elzinga et al., 2016; Nugent, Sumner, & Amstadter, 2014) the present special issue of the journal brings together a collection of four papers from some of the most impactful and innovative ideas that were presented in Dallas.

Considering the conference theme, the first paper in this special section, authored by Dr Kessler and colleagues (Kessler et al., 2017), represents a major contribution to acknowledging the global public health burden of PTSD as assessed by the World Mental Health Surveys. Their study represents the largest epidemiological research of PTSD status published to date, carried out in face-to-face home-based interviews of 68,894 participants from 24 different countries ranging in economic status across low/lower-middle income (People’s Republic of China, Colombia, Nigeria, Peru, Ukraine), upper-middle income (Brazil, Bulgaria, Colombia, Lebanon, Mexico, Romania, South Africa) and high income (Australia, Belgium, France, Germany, Israel, Italy, Japan, Netherlands, New Zealand, Northern Ireland, Portugal, Spain, U.S.A.). Here, conditional risk for PTSD was determined in the 70% of these respondents (numbering over 50,000) who reported that they had experienced one or more traumatic life events from one or more of seven different event categories (i.e. those relating to war [13%], physical violence [23%], intimate partner or sexual violence [14%], accidents [34%], those involving the unexpected or traumatic death of a loved one [36%], other traumas that happened to other people [36%] and those otherwise unclassified [8%]). Importantly, Kessler et al. (2017) report that nearly 75% of respondents indicated having experienced more than a single type of traumatic event, with the mean number of lifetime trauma types experienced reported to be nearly five (4.6) once the mean number of exposures to different trauma types was taken into account in statistical analyses. They further confirmed prior trauma exposure as a risk factor for subsequent exposure, both within and across trauma types.

Considering the prevalence of multiple trauma exposure, an important study goal for Kessler et al. (2017) was to determine the conditional risk for PTSD to randomly selected categories of events among multiply exposed persons, in order to address the bias intrinsic to the more common method of assessing conditional risk in response to the category of events deemed ‘the worst or most upsetting’ by the respondent. Utilizing this novel assessment-analytic strategy, Kessler et al. report that PTSD varied significantly by trauma type. Thus, whereas PTSD risk in trauma-exposed persons was found to be approximately 4% across event categories, it was over 11% for the highest-risk event category, namely events involving intimate partner or sexual violence, where they replicated the common finding that approximately one in five persons (19%) who report that they have been raped can be expected to develop PTSD. Indeed, the authors describe rape and other sexual assault as among the most frequent causes of PTSD, together accounting for approximately 18% of PTSD cases. Still, considering the high frequency with which people experience the unexpected death of loved ones, the authors note that it is actually this class of events that by far represents the most frequent single cause of PTSD within the general population, alone accounting for a nearly 3% PTSD prevalence and over 22% of all lifetime PTSD episodes. The authors also investigated a number of sociodemographic predictors of trauma exposure, where gender, marital status, level of education and age were replicated as well-known risk factors, but were emphasized as differentially predicting risk across event categories. For example, whereas across trauma types, risk for trauma exposure was reported to be highest during childhood-adolescence and later in life (among those 65 years of age or older), risk of interpersonal violence was found to be highest among late adolescents aged 17–18, while risk for accidents and unexpected death of loved ones was highest in early adulthood (median ages-of-occurrence 24–31).

Kessler et al. (2017) further characterized the typical course of PTSD symptoms within this large population-based sample, giving consideration to the global social and economic burden of trauma exposure and PTSD. The authors found that whereas 25–40% of PTSD cases can be expected to remit within one year, recovery among the majority of persons can be expected to require longer than this. Specifically, the authors report that mean duration of symptoms is approximately six years across trauma types, with the most chronic cases evidenced for combat-related PTSD, the latter associated with a mean duration of over 13 years. Taking the often chronic-course of PTSD into consideration, the authors thus report that the lifetime burden of PTSD amounts to nearly 78 person-years for every 100 participants assessed. However, again taking into account that trauma exposure rates varied significantly by event category, the authors further report that the broad category of intimate partner-sexual violence accounts for the majority of the burden attributable to PTSD in person-years, at approximately 43% of the cumulative burden. The unexpected death of a loved one, by comparison, also alone accounts for nearly 12% of such burden.

Collectively Kessler and colleagues’ findings make the enormity of the global social and economic burden of trauma exposure and PTSD simply unavoidable. This is the more true if we realize that stressful events not fulfilling the strict A1 criterion may give rise to similar or even higher rates of PTSD than A1 events (e.g. Van Den Berg, Tollenaar, Spinhoven, Penninx, & Elzinga, 2017). As the authors themselves discuss, their study findings represent a call to action. In fact, they clarify that their surveys were expressly designed as needs assessments in order to aid government policy planning to better recognize the prevalence and outcomes of trauma exposure and PTSD in their respective nations. Acknowledging that treatment seldom meets even minimal standards of adequacy internationally, they recommend outreach efforts to address the magnitude of trauma exposure and PTSD as a truly global public health problem.

Whereas Kessler and colleagues’ study primarily emphasizes social and psychological determinants of risk for trauma exposure and PTSD, the second paper of this special issue by Dr Binder (2018) summarizes research aimed at understanding the molecular mechanisms underlying gene by environment interactions with an emphasis on the glucocorticoid (GC) system and its modulation by *FKBP5* genetic variants (see also Fiori & Turecki, 2016; Lanius & Olff, 2017). As is commonly known (e.g. Sapolsky, 2004), the GC system is activated in response to threat, where the release of ACTH from the pituitary triggers GC secretion from the adrenal gland into systemic blood flow, thus modulating responsiveness when finding contact at mineralocorticoid (MR) and glucocorticoid receptors (GR) distributed throughout brain and body. Dr Binder describes both MR and DR as cytoplasmic receptors that translocate to cell nuclei when activated, thus serving as transcription factors that bind to specific DNA sequences (so-called glucocorticoid responsive elements or GREs), thereby either enhancing (up-regulating) or suppressing (down-regulating) transcription of a larger number of genes. Beyond this, Dr Binder notes that epigenetic variation also influences the response to transcription factors through post-translational modifications of histone proteins as well as chemical modifications of single nucleotides, thereby modifying the accessibility of the DNA to transcriptional regulators (given that more condensed chromatin is more prohibitive for binding of GRs and transcriptional regulation of target genes). Thus, Dr Binder relates that GR activation is able to induce lasting epigenetic changes at GRE by locally decreasing the level of DNA methylation, which facilitates the transcriptional effects of the GR on the target genes and, under excessive GC release in cases of high or chronic stress exposure, could induce long-lasting epigenetic changes.

However, Dr Binder further describes that the aforementioned epigenetic responses to GC are likely in turn further moderated by other genetic variants, using *FKBP5* as an exemplar case. She notes the central role played by *FKBP5* in the stress response, first in that when bound to the GR complex it downregulates the affinity of the GR to cortisol and prevents its ready translocation to the nucleus, and second in that it is itself a target of GR activation, where its mRNA and protein are induced by cortisol, creating an ultra-short negative feedback loop. Moreover, Dr Binder describes *FKBP5* as not only regulating GR function but additionally many other proteins and pathways broadly implicated in neuronal function and synaptic plasticity, thus amplifying the stress response presumably in a cell-type-specific manner. She therefore relates that variation in *FKBP5* expression (in turn by genetic or epigenetic factors) is likely to have demonstrative consequences for stress-related behaviour. Indeed, she relates that *FKBP5* mRNA is known to be moderated by common genetic variants where approximately 65% of humans carry an allele associated with a moderate increase in *FKBP5* following GR activation, while the remaining 35% carry an allele associated with a more exaggerated mRNA response. She notes that the results of such genetic variants partly moderate neuroendocrine (e.g. prolonged cortisol release), behavioural (e.g. selective attention allocation) and subjective (e.g. intrusions, dissociative) responses to stress.

Given this background, it is perhaps not surprising that interactions between *FKBP5* genotype and exposure to stressful and traumatic life events, particularly those experienced during childhood, should represent risk factors for psychological disorders including PTSD. However, in the particularly interesting case of *FKBP5*, Dr Binder teaches that genetic and epigenetic changes themselves interact, where reduction in DNA methylation of *FKBP5* locus GREs is thought to lead to an enhancement of the transcriptional response of FKBP5 to GC only among persons with exposure to child abuse. She concludes with the hope that further knowledge into the molecular mechanisms underlying differential genetic risk for PTSD (and related psychological disorders in response to trauma exposure) will result in biomarkers that will aid effective treatment. For example, she relates that such knowledge could help predict the likelihood of a full recovery in response to existing psychological treatments, or even lead to the development of approaches to selectively inhibit *FKBP5* function directly following or immediately before the onset of a potentially traumatic event.

In the third paper in this series, Rizzo and Shilling (2018) describe their application of innovative technology to psychological intervention for PTSD and related disorders, namely virtual reality (VR) exposure therapy (VRET) (e.g. additional reviews by Gonçalves, Pedrozo, Coutinho, Figueira, & Ventura, 2012; Motraghi, Seim, Meyer, & Morissette, 2014), focusing particularly on their own work in developing the BRAVEMIND VRET system. As Rizzo and Shilling note, although VR is not a particularly new technology, with initial demonstrations taking place as early as the 1980s, recent advances in both hardware and software have made possible increasingly life-like engagement with increasingly realistic simulated ecological environments. In brief, within the context of exposure therapy for PTSD, VRET makes possible the presentation of trauma-relevant stimuli in immersive and dynamic three-dimensional visual-auditory displays by which users can engage with, effectively facing and overcoming, stimuli reminiscent of long held fears. In some ways, VRET represents an interesting midway point between imaginal and in vivo exposure wherein, from the perspective of exposure therapy, it is a particularly relevant treatment modality in cases when opportunities for in vivo exposure exercises are unavailable or impractical (e.g. referring to warzone fears in persons with combat-related PTSD). Rizzo and Shilling note that VRET may also offer some specific advantages over imaginal exposure, for example, by delivering realistic multi-sensory stimulation, and overcoming both patients’ tendencies toward avoidance and any limitations inherent to their imagination and memory for trauma context-relevant cues. The authors review some of the promising initial trials of the BRAVEMIND and other VRET systems, including case studies, open trials and randomized clinical trials, the latter in comparison with waitlist, treatment as usual, or imaginal exposure and sometimes either as augmenting or as augmented by typical evidence-based psychological and pharmacological treatments. Recent VRET studies have focused not only on software relevant to direct combat trauma, but also on the kinds of traumatic events more often experienced by combat medics/corpsmen, as well as events involving military sexual trauma.

Rizzo and Shilling (2018) also describe clinical uses for VR technology beyond exposure therapy, for example, in psychological and psychophysiological assessment and resilience training. Coupled with developments in artificial intelligence (AI), they further describe the potential application of virtual human agents presented within VR environments who are able to perform basic clinical services, for example, clinical interviewing or providing psychoeducational interventions. As an example, Rizzo and Shilling describe in some detail their SimCoach and SimSensei virtual human projects, involving a responsive and non-verbally sensitive AI-based virtual humans capable of collecting background clinical information through the administration of screening questionnaires and interview techniques, as well as providing clinical advice and support, including directing users to relevant online information in order to facilitate the process of seeking appropriate clinical care. Rizzo and Shilling (2018) conclude in making their case for why VR may address barriers to clinical care among traumatized persons in the future. Specifically, the authors refer to ‘7 A’s’ as reasons why they believe that VR will be increasingly relevant to clinical practice for PTSD in the future: raising Awareness, Anticipated Benefit from patients, increasing Accessibility, increasing Availability of well-trained providers, recognized Acceptability of seeking treatment, ease of Adherence and increasing Affordability of VR technology.

It has been an emerging and successful tradition at the ISTSS meeting (compare Yehuda et al., 2016) to gather experts together for a frank discussion about approaches that have stood the test of time, in other words tried and true evidence-based practices (EBPs), in comparison with others approaches that can by now be considered more or less tried and failed. In the final paper in this special issue, Schnurr, Bryant, Berliner, Kilpatrick, Rizzo, and Ruzek (2017) continue this line of discussion, describing some of the things they have changed their mind about over the course of their careers, primarily from the perspectives of EBPs, public health and technology.

First, Dr Schnurr opens this discussion by describing her increasing recognition of the variable measures by which EBPs for PTSD work. In other words, while she makes clear that overall EBPs *do* work, she also acknowledges that how *well* they work remains an important research question, and she acknowledges the need for continued improvement in PTSD treatment. As one such effort from a technology standpoint, she briefly describes research to develop an online decision aid in order to enhance patients’ knowledge about effective treatment options. Second, Dr Bryant describes his increasing recognition of the need to scale down EBPs if they are to be feasible public health strategies in poorly resourced settings, including having shorter treatments delivered by lay providers in stepped care frameworks. Third, Dr Berliner also considers how global access to trauma-focused treatments such as trauma-focused cognitive-behavioural therapy and other EBPs could be increased through creative, low cost approaches. She makes some specific recommendations, for example, encouraging training methods that cover more than one EBP derived from similar theories and contain similar specific interventions, giving greater consideration to transdiagnostic interventions, and utilizing some common methods across EBPs for example in treatment monitoring and progress notes. Fourth, Dr Kilpatrick describes his increasing recognition of some of the limits to the impact of scientific methodology in guiding treatment outcome and implementation research for trauma- and stressor-related disorders. Specifically, he laments that research findings have not had as significant an impact on public opinion, public policy, and clinical practice than he might have by now expected since beginning in the field in the mid 1970s. He attributes this shortcoming partly to researchers being somewhat unsuccessful in translating their findings into practice, or making clear the real life implications of their results. However, he also acknowledges that the many problems and complex needs of traumatized persons are simply not fully addressed by even the best of EBPs, and he advises that a public health perspective will likely be needed if we are to better take up the burden of helping traumatized persons fully recover. Fifth, Dr Rizzo describes that whereas he might have once believed exposure therapies were alone sufficient for PTSD treatment, he now believes that varied, stepwise, and multicomponent approaches to treatment will be required if are we are to effectively treat the multitude of presenting problems that can occur following traumatic stress. Included within such an expanded list of treatment options, Dr Rizzo considers whether interventions from the broad field of complementary and alternative medicine (on which this journal currently has a call open: click here) may be of application in PTSD recovery, and reconsiders the potential role of VR technology in PTSD treatment. He also recommends more research into the barriers of receiving care for PTSD, including perceptions of anticipated benefits, accessibility and acceptability of current EBPs. Finally, Dr Ruzek also considers how the application of technology might address barriers to care for traditional face-to-face individualized EBPs for PTSD treatment. In particular, he describes some of his research exploring Internet- and telephone-based approaches as means of reducing the burden of training clinicians as providers of EBPs. He also considers the potential of novel technologies in providing direct treatment, monitoring treatment outcomes and aiding clinician self-care. See also the call for papers on eHealth (click here).

In summary, this collection of four papers collected from the meeting of the ISTSS in 2016 begins to describe the burden of traumatic stress as a truly global public health problem. The present special section is a treasure trove of ideas collected from experts in traumatic stress studies that, put in place, should help us move forward in finding a solution.

## References

[CIT0001] Binder E. B. (2018). Dissecting the molecular mechanisms of gene x environment interactions: implications for diagnosis and treatment of stress-related psychiatric disorders. *European Journal of Psychotraumatology*, 8(sup5), 1412745. doi:10.1080/20008198.2017.1412745 29372006 PMC5774411

[CIT0002] Elzinga B., Schmahl C., & Olff M. (2016). Back to basics: Integrating clinical and scientific knowledge to advance the field of Trauma—Highlights of the ISTSS-2015. *European Journal of Psychotraumatology*, 7(s2). doi:10.3402/ejpt.v7.33765 PMC5106863 27837584

[CIT0003] Fiori L. M., & Turecki G. (2016). Investigating epigenetic consequences of early-life adversity: Some methodological considerations. *European Journal of Psychotraumatology*, 7. doi:10.3402/ejpt.v7.31593 PMC5106862 27837582

[CIT0004] Gonçalves R., Pedrozo A. L., Coutinho E. S. F., Figueira I., & Ventura P. (2012). Efficacy of virtual reality exposure therapy in the treatment of PTSD: A systematic review. *PLoS ONE*, 7(12), e48469. doi:10.1371/journal.pone.0048469 23300515 PMC3531396

[CIT0005] Kessler R. C., Kessler R. C., Aguilar-Gaxiola S., Alonso J., Benjet C., Bromet E. J., ... Koenen K. C. (2017). Trauma and PTSD in the WHO world mental health surveys. *European Journal of Psychotraumatology*, 8(1), 1314165. doi:10.1080/20008198.2017.1353383 PMC5632781 29075426

[CIT0006] Lanius R., & Olff M. (2017). The neurobiology of PTSD. *European Journal of Psychotraumatology*, 8(1), 1314165. doi:10.1080/20008198.2017.1314165 PMC5475317 28649299

[CIT0007] Magruder K. M., McLaughlin K. A., & Elmore Borbon D. L. (2017). Trauma is a public health issue. *Journal of Psychotraumatology*, 8(1), 1375338. doi:10.1080/20008198.2017.1375338 PMC5800738 29435198

[CIT0008] Motraghi T. E., Seim R. W., Meyer E. C., & Morissette S. B. (2014). Virtual reality exposure therapy for the treatment of posttraumatic stress disorder: A methodological review using CONSORT guidelines. *Journal of Clinical Psychology*, 70, 197–6. doi:10.1002/jclp.22051 24108479

[CIT0009] Nugent N. R., Sumner J. A., & Amstadter A. B. (2014). Resilience after Trauma: from surviving to thriving.*European Journal of Psychotraumatology*, 5. doi:10.3402/ejpt.v5.25339 PMC4185140 25317260

[CIT0010] Rizzo A., & Shilling R (2018). Clinical virtual reality tools to advance the prevention, assessment, and treatment of ptsd. *European Journal of Psychotraumatology*, 8(sup5), 1414560. 10.1080/20008198.2017.1414560 PMC5774399 29372007

[CIT0011] Sapolsky R. M. (2004). *Why zebras don’t get ulcers* (3 ed.). New York, NY: Holt Paperbacks.

[CIT0012] Schnurr P. P., Bryant R., Berliner L., Kilpatrick D. G., Rizzo A., & Ruzek J. I (2017). What I have changed my mind about and why: Public health and technology perspectives in the field of trauma studies. *European Journal of Psychotraumatology*, 8(sup5), 1372007. doi: 10.1080/20008198.2017.1372007 29075427 PMC5632765

[CIT0013] van den Berg L. J. M., Tollenaar M. S., Spinhoven P., Penninx B. W. J. H., & Elzinga B. M. (2017). A new perspective on PTSD symptoms after traumatic vs stressful life events and the role of gender. *European Journal of Psychotraumatology*, 8(1), 1380470. doi:10.1080/20008198.2017.1380470 PMC5800737 29435199

[CIT0014] Yehuda R., Spiegel D., Southwick S., Davis L. L., Neylan T. C., & Krystal J. H (2016). What I have changed my mind about and why. *European Journal of Psychotraumatology*, 7. doi:10.3402/ejpt.v7.33768 PMC5106864 27837585

